# Shockwaves Inhibit Chondrogenic Differentiation of Human Mesenchymal Stem Cells in Association with Adenosine and A2B Receptors

**DOI:** 10.1038/s41598-017-14875-y

**Published:** 2017-10-30

**Authors:** Lei Tan, Bin Zhao, Fu-Tao Ge, Da-Hui Sun, Tiecheng Yu

**Affiliations:** 1 Department of Orthopedic Trauma, The first Hospital of Jilin University, Changchun, 130021 China; 2 Department of Orthopedics, The Bin Zhou People’s Hospital, Bin Zhou, 256600 China; 3 Department of Shockwave, The First Hospital of Jilin University, Changchun, 130021 China

## Abstract

Extracorporeal shockwave therapy (ESWT) has emerged as the important choice for the treatment of many orthopedic disorders. Our previous mechanistic studies suggest that ESWT promoted osteogenesis of human mesenchymal stem cells (hMSCs) through mechanisms that involve adenosine 5′-triphosphate (ATP) release. In this study, we investigated the effect of ESWT on chondrogenesis of hMSCs. We demonstrate that ESWT treatment caused a significant release of adenosine from hMSCs; ESWT treatment increased the levels of A2B receptor (A2BR) in hMSCs under 3-D culture conditions. ESWT, exogenous adenosine and specialized A2BR agonist suppressed hMSC chondrogenic differentiation through downregulating the expressions of aggrecan (ACAN), Collagen Type I alpha 2(COL1A2), Collagen Type II alpha 1(COL2A1), Sex-Determining Region YBox 9 (SOX9) and Sex-Determining Region YBox 6 (SOX6). Selective A2BR antagonists induced chondrogenic differentiation of hMSCs. This study indicated that shockwave therapy inhibits hMSC chondrogenic differentiation through or partially through regulation of adenosine release and activation of A2B receptor under 3-D culture conditions.

## Introduction

Cartilage is a highly organized, avascular connective tissue that provides low friction, shock absorbance, load bearing and distribution for effective joint movement. Cartilage defects cause joint pain, stiffness and loss of mobility due to osteoarthritis, trauma, aging, and developmental disorders^1^. Due to the limited regenerative capacity of chondrocyte, the cartilage defects cannot fully repair itself^2^. Although methods including subchondral drilling, osteochondral allograft, and periosteal or perichondral tissue grafting have been attempted to repair defects in articular cartilage, these techniques cannot reproduce the reparative tissue characteristics of hyaline cartilage for optimal filling of the defects^3^.

Mesenchymal stem cells (MSCs) are able to self-replicate and differentiate into a variety of cell types such as osteoblasts, chondrocytes, adipocytes, and smooth muscle cells^4^. Differentiated MSC producing hyaline cartilage tissue have shown promising early results in the repair of localized cartilage defects and may provide further treatment approaches for more extended cartilage damages in the future^5^.

In the past decades, extracorporeal shockwave therapy (ESWT) had emerged as the important choice in the treatment of many orthopedic disorders including shoulder pathology, chronic diabetic foot ulcers, osteoarthritis of the knee, chronic patellar tendinopathy, bone nonunion and avascular necrosis of the femoral head^6–11^. However, the exact mechanisms of shockwave therapy in musculoskeletal healing is still unknown^12^. Our recent study suggested that the mechanism of shockwave promoting osteogenesis may be related to adenosine 5′-triphosphate (ATP)^13^. However, ATP cannot survive outside the cell, and is hydrolyzed into adenosine in a short time. Therefore, adenosine may be an important factor in the biological effects of shockwaves.

Adenosine in the extracellular compartment functions as a signaling molecule through the activation of G-protein-coupled receptors. Four distinct adenosine receptors (ARs) have been described, A1, −2A, -2B, and -3, which differ in their affinity for adenosine and the biological effects of their associated signaling pathways^14^. The inhibition of A1 and A3 ARs and the activation of A2A and A2B ARs adenylyl cyclase causing decreases and increases in the intracellular concentration of Cyclic Adenosine monophosphate (cAMP), respectively^15^. The four ARs are widely involved in neurodegenerative, immune, cardiac, inflammatory disorders, cancer and bone homeostasis^16,17^. A2B receptor (A2BR) is the most insensitive AR of the four^14^. Its engagement stimulates osteoblast differentiation of MSC precursors^3,4,6–8,13^. In addition, A2BR occupancy suppresses osteoclast differentiation and function as well^3^. A2BR signaling pathway can stimulate the production of IL-6, which may have an important role in the differentiation of MSCs.

In this study, we investigated the effect of shockwave treatment on hMSCs chondrogenesis and explored the potential mechanisms. Our data showed that shockwave treatment released significant amounts of adenosine from hMSCs. Exogenous adenosine and specialized A2B agonist suppressed hMSC chondrogenic differentiation through downregulating the expressions of ACAN, COL1A2, COL2A1, SOX9 and SOX6. Selective A2B antagonists induced chondrogenic differentiation of hMSCs.

## Results

### Shockwave treatment increases the release of ATP, ADP, AMP, and adenosine from hMSCs

**Figure 1:**
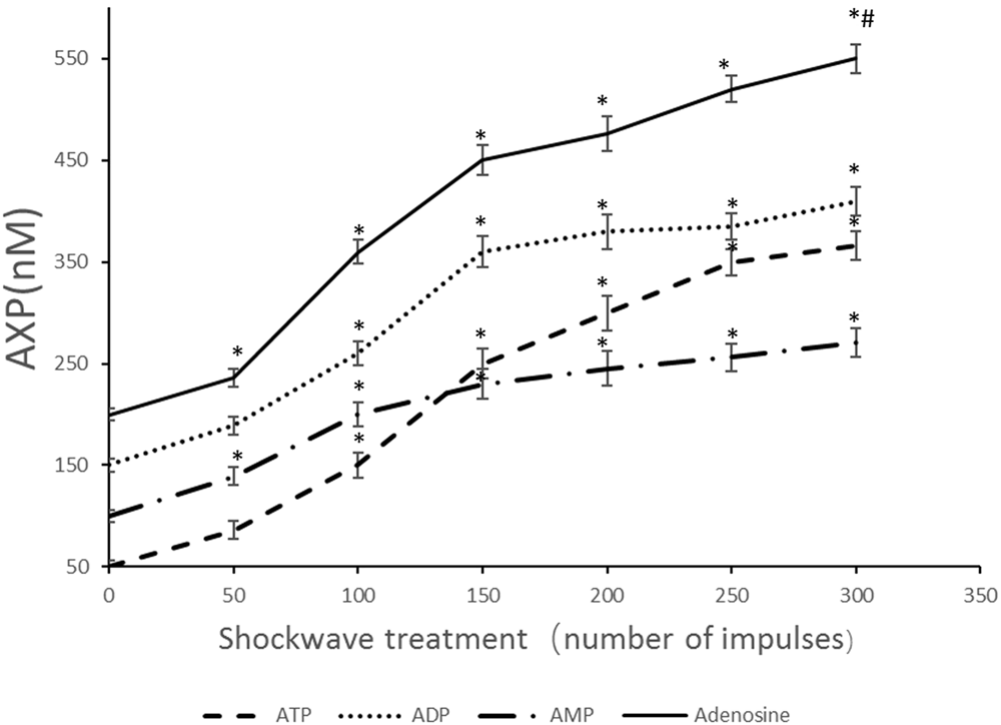
The titer of extracellular concentration of AXP (ATP, ADP, AMP and Adenosine) was detected by enzyme-linked immunosorbent assay (ELISA). Passages 4 hMSCs (5 × 10^5^ per ml) were allowed to rest for 3 hours at 37 °C. Then the cells were exposed to shockwave treatment (0.18 mJ/mm^2^) using the indicated impulse numbers. The titer of extracellular concentration of AXP was determined 1 h after shockwave treatment; Data are expressed as mean ± SD. Asterisks indicate statistically significant differences compared to the control group (cells not subjected to shockwave treatment) *p < 0.01; Hashes indicate statistically significant differences compared to cells subjected to 200 impulses shockwave treatment; ^#^p < 0.01; Student’s t test.

MSCs was treated once with SW (0.18 mJ/mm^2^) using the increased impulse numbers, then subject to 3D chondrogenic differentiation for 1 hour, and the levels of ATP, ADP, AMP and adenosine were evaluated by ELISA. There were no significant differences between groups at baseline. The concentration of adenosine as well as ATP, ADP, AMP in shockwave treated group dramatically increased from 0 to 150 impulses in a dose-dependent way (Fig. 1). For the treatment group of impulses more than 150, the results were more confused. Differential trend of ATP, ADP, AMP, and adenosine was apparent. There were no significant differences for ATP, ADP and AMP compared with the 200 impulses group (Fig. 1), while the release of adenosine increased significantly (p < 0.01).

### Shockwave exerts differential influence on the viability of hMSCs

**Figure 2:**
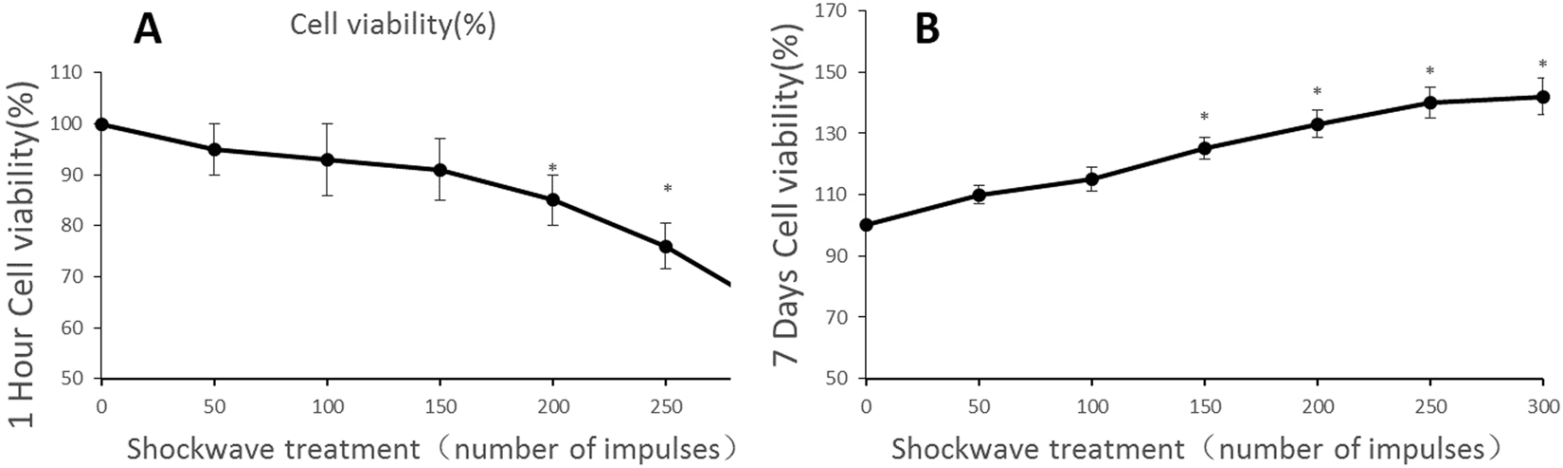
Effect of shockwaves on the viability of hMSCs. (**A**) After four passages, hMSCs (5 × 10^3^ per ml) were exposed to shockwaves using the indicated impulse numbers. Cell viability was determined 1 h after shockwave treatment; (**B**) Cell viability was determined 7 days after shockwave treatment (data was shown in mean ± SD percentages of control from independent experiment, n = 3, *p < 0.05; Student’s t test).

MSCs was treated once with SW (0.18 mJ/mm^2^) using the increased impulse numbers, then subject to 3D chondrogenic differentiation for 1h and 7 days, and the viability of MSCs was evaluated by via an CCK-8 assay after 1 h incubation. In this set of experiments, we investigated the effect of shockwave under three-dimensional (3D) culture conditions for 1 h and 7 days on. a. Shockwave reduced cell viability in the short term: Viability of cells subjected to <200 shockwave impulses remained at >95% when examined immediately after shockwave treatment (Fig. 2A). However, cells exposed to 200 impulses showed significantly decreased viability after one hour (Fig. 2A). b. Shockwave promoted cell viability in the long term: As shown in Fig. 2B, cell viability was enhanced in hMSCs dose-dependently. There was only slight effect at 50 impulses, but a significant increase in viability was observed in the cells treated with 150 and above impulses compared to 0 impulses. In groups with 200 and above impulses, no significant difference was found compared with cells treated with 150 impulses shockwave (p > 0.05).

### Shockwave treatment increased the levels of A2BR in hMSCs under 3-D culture conditions

**Figure 3:**
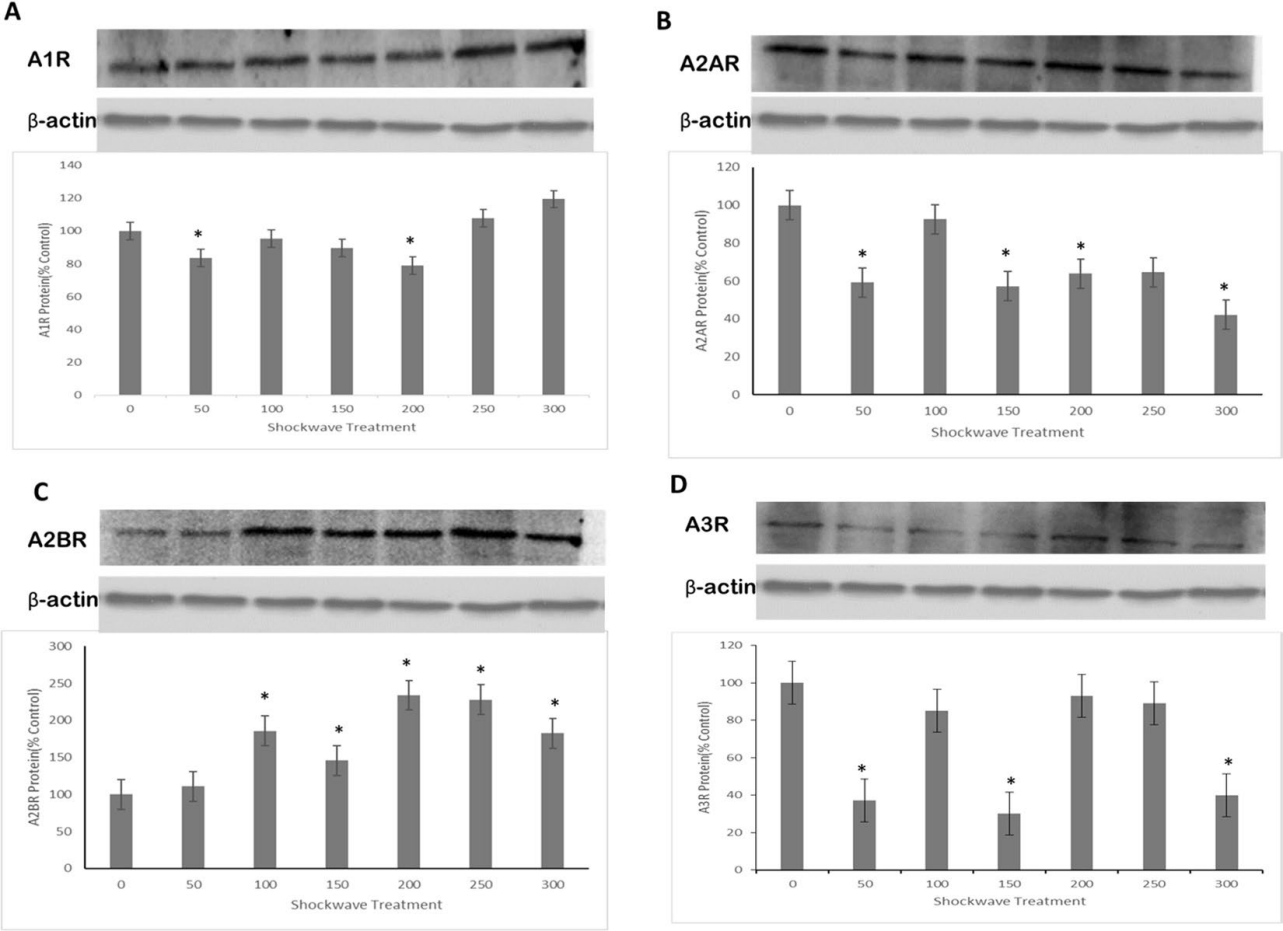
Expressions of four subtypes of adenosine receptors during chondrogenesis in hMSCs pellet cultures after shockwave treatment were evaluated by western blotting. Passages 4 hMSCs (5 × 10^5^ per ml) were exposed to shockwaves using the indicated impulse numbers, were rested for 60 minutes. Four subtypes of AR expression were detected in shockwave treated hMSCs pellet culture for chondrogenesis induction for 21 days. β-actin was used as an internal control. The sizes of the protein bands were as follows: A1R:38 kDa, A2AR:46 kDa, A2BR:52 kDa, A3R:34 kDa and β-actin:43 kDa, and data were normalized by control non-treated group and present in the corresponding graphs. Data were representative from three independent experiments.

**Figure 4:**
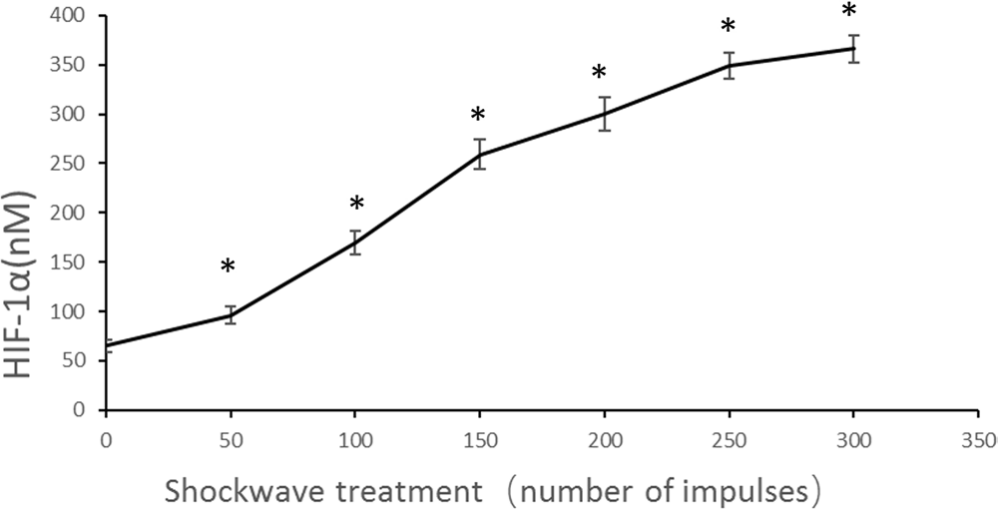
The titer of extracellular concentration of hypoxia-inducible factor-1α (HIF-1α) was detected by enzyme-linked immunosorbent assay (ELISA). Passages 4 hMSCs (5 × 10^5^ per ml) were allowed to rest for 3 hours at 37 °C. Then the cells were exposed to shockwave treatment (0.18 mJ/mm^2^) using the indicated impulse numbers. The titer of extracellular concentration of HIF-1α was determined 1 h after shockwave treatment; Data are expressed as mean ± SD. Asterisks indicate statistically significant differences compared to the control group (cells not subjected to shockwave treatment); *p < 0.05; Student’s t test.

MSCs was treated once with SW (0.18 mJ/mm^2^) using the increased impulse numbers, then subject to 3D chondrogenic differentiation for 21 days, and the expression of the four known adenosine receptors (A1R, A2AR, A2BR, A3R) in MSCs were evaluated by Western blotting (Fig. 3). A2BR was significantly increased in MSCs following 100, 150, 200, 250 impulses treatment with shockwave. After mechanical stimulation of MSCs, A2BR was up-regulated by a mean factor of 0.41. No consistent change was noticed between different groups in the levels of A1R, A2AR and A3R. This suggested that A2BR was possibly involved in the chondrogenesis effects of shockwave treatments in MSCs under 3D culture environment. The levels of HIF-1α were evaluated by ELISA. The concentration of HIF-1α in shockwave treated group dramatically increased from 0 to 150 impulses in a dose-dependent way (Fig. 4).

### Shockwave treatment inhibits chondrogenic differentiation of hMSCs under 3D culture conditions

**Figure 5:**
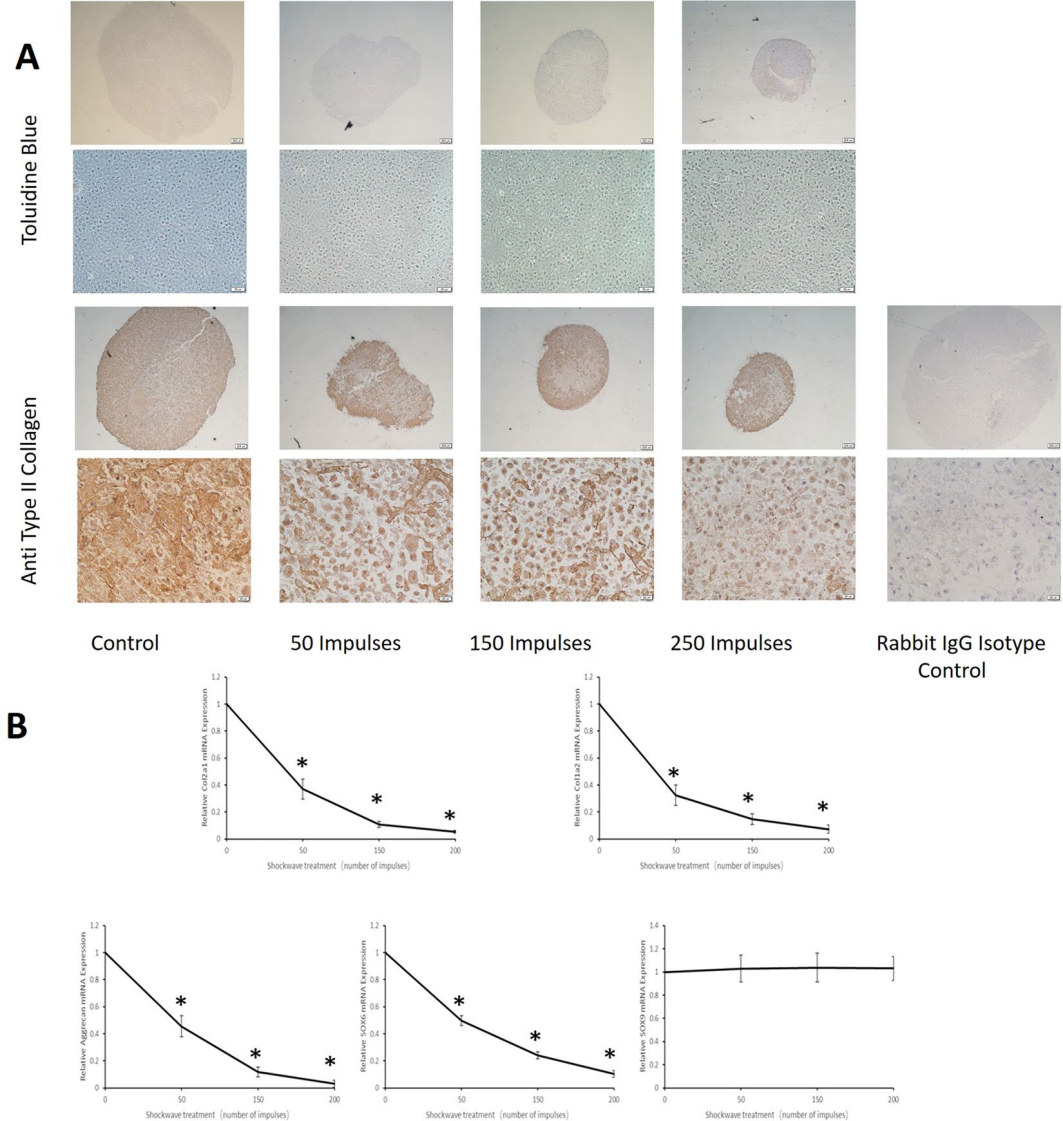
Shockwave treatment inhibits chondrogenic differentiation of hMSCs. Passages 4 hMSCs (5 × 10^5^ per ml) were exposed to shockwaves using the indicated impulse numbers and followed by 21 days of chondrogenic differentiation. (**A**) Pellets were stained with toluidine blue or anti–type II collagen antibody or rabbit isotype IgG control antibody. Results in A are representative of 3 independent experiments with similar findings. (**B**) Relative gene expressions for SOX6, SOX9, COL1A2, COL2A1 and ACAN in pellet culture were determined by real-time PCR. Values are the mean ± SD (n = 3). *p < 0.05 in comparison to the control group (cells not subjected to shockwave treatment).

One of the main objectives of the present observational study was to investigate the effect of shockwave treatment on hMSC chondrogenic differentiation in 3D pellet culture at both mRNA and protein levels. MSCs was treated once with SW (0.18 mJ/mm^2^) using the increased impulse numbers, then subject to 3D chondrogenic differentiation for 21 days. Upon treatment of shockwave, a suppressed accumulation of the cartilage matrix was detected by toluidine blue staining and immunostaining with anti–type II collagen antibody (Fig. 5A). Gene expression levels of the differentiated cells were assessed for hyaline cartilage markers COL2A1, SOX9, SOX6 and ACAN, fibrocartilage marker (collagen type I) and the internal control marker of glyceraldehyde-3-phosphate dehydrogenase (GAPDH).

There was a suppressed chondrogenic response with changes in gene expression in cells treated by 0, 50, 150 and 250 impulses of shockwave (Fig. 5B). The expressions of SOX6, collagen types II and I, and ACAN were significantly decreased by 19-, 12-, 7-, and 6-fold, respectively (p < 0.05).

### Shockwave treatment inhibits chondrogenic differentiation of hMSCs through or partially through the adenosine receptor A2B

MSCs was treated once with SW (0.18 mJ/mm^2^ 150 impulses), or exogenous adenosine (100 μM), then subject to 3D chondrogenic differentiation for 7 and 21 days, in absence or presence of the indicated agents (PSB115: 1 μM; BAY60-6583: 1 μM), and relative gene expressions of SOX6, SOX9, COL1A2, COL2A1 and ACAN were determined by real-time PCR.

**Figure 6:**
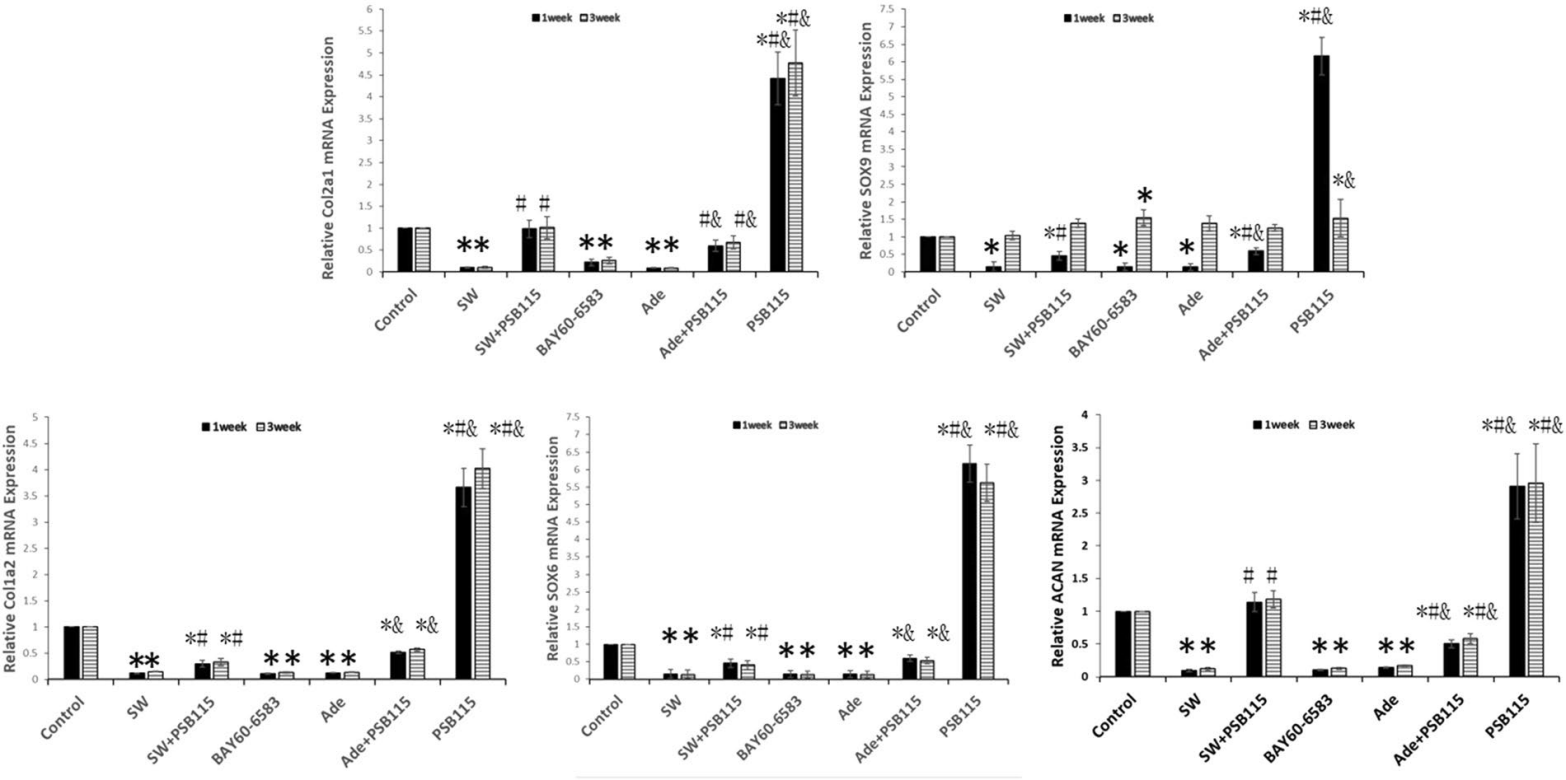
Shockwave treatment inhibits chondrogenic differentiation of hMSCs possibly through the A2BR. MSCs was treated once with SW (0.18 mJ/mm^2^ 150 impulses), or once with exogenous adenosine (100 μM), then subject to 3D chondrogenic differentiation for 7 and 21 days, in absence or presence of the indicated agents (PSB115: 1 μM; BAY60-6583: 1 μM). Relative gene expressions of SOX6, SOX9, COL1A2, COL2A1 and ACAN in pellet culture were determined by real-time PCR. Relative expression of the targeted gene was presented as a ratio to control group of the respective time point. Asterisks indicate statistically significant differences compared to cells not subjected to shockwave treatment; *p < 0.05; Hashes indicate statistically significant differences compared to cell subjected to 150 impulses shockwave treatment; ^#^p < 0.05; And indicate statistically significant differences compared to cells subjected to adenosine treatment; ^&^p < 0.05; Student’s t test. Values are the mean ± SD. Abbreviations: SW, shockwave; Ade, Adenosine.

The adenosine or agonist of A2BR can simulate the effect of shockwave. While pellets were treated by adenosine or A2BR specific agonist BAY60-6583, there was no significant difference in the gene expression of SOX6, COL1A2, COL2A1 and ACAN compared with the shockwave group (Fig. 6; p > 0.05). The antagonist of A2BR can inhibit the effect of shockwave. While pellets were treated by A2BR specific antagonist PSB115, there was significant increase in the gene expressions of SOX6, COL1A2, COL2A1 and ACAN compared with control group (p < 0.01). While the combination of shockwave treatment and PSB115 resulted significant counteracted effect in the gene expression of SOX6, COL1A2, COL2A1 and ACAN compared with shockwave group (Fig. 6; p < 0.01).

### Shockwave treatment inhibits chondrogenic differentiation of hMSCs possibly related with IL-6

**Figure 7:**
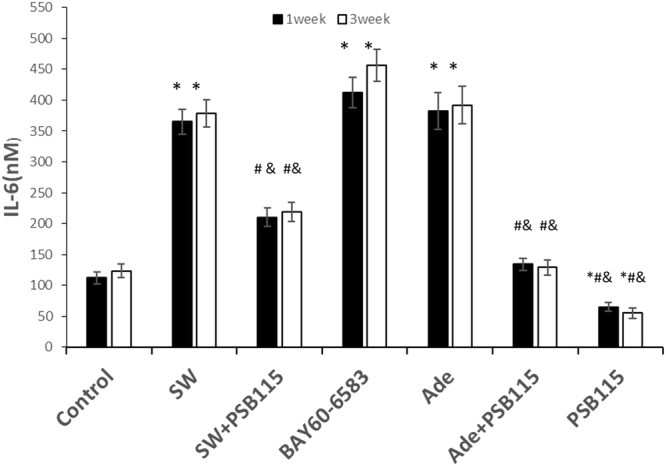
Shockwave treatment inhibits chondrogenic differentiation of hMSCs possibly related with IL-6. MSCs was treated once with SW (0.18 mJ/mm^2^ 150 impulses), or once with exogenous adenosine (100 μM), then subject to 3D chondrogenic differentiation for 7 and 21 days, in absence or presence of the indicated agents (PSB115: 1 μM; BAY60-6583: 1 μM). The extracellular concentration of IL-6 was detected by enzyme-linked immunosorbent assay (ELISA). Cells were allowed to rest for 3 hours at 37 °C before they were treated with indicated agents. Asterisks indicate statistically significant differences compared to cells not subjected to shockwave treatment; *p < 0.05; Hashes indicate statistically significant differences compared to cell subjected to 150 impulses shockwave treatment; ^#^p < 0.05; And indicate statistically significant differences compared to cells subjected to adenosine treatment; ^&^p < 0.05; Student’s t test. Values are the mean ± SD. Abbreviations: SW, shockwave; Ade, Adenosine.

MSCs was treated once with SW (0.18 mJ/mm^2^ 150 impulses), or exogenous adenosine (100 μM), then subject to 3D chondrogenic differentiation for 7 and 21 days, in absence or presence of the indicated agents (PSB115: 1 μM; BAY60-6583: 1 μM), and the extracellular concentration of IL-6 was detected by enzyme-linked immunosorbent assay (ELISA). The shockwave treatment increased the secretion of IL-6, while the agonist and antagonist of A2BR promoted and inhibited the secretion of IL-6 (Fig. 7; p < 0.05). This revealed that shockwaves might play a role in regulating the effect of chondrogenic differentiation through the A2BR-IL-6 signaling pathway.

## Discussion

The clinical application of shockwave in the treatment of nonunion and necrosis of the femoral head has achieved good results^18–20^. Our previous study showed that shockwaves released cellular ATP that activated P2 × 7 receptors and downstream signaling events and promoted osteogenic differentiation of hMSCs^13^. However, there are few studies investigating the effect of shockwaves on the cartilage regeneration. In this study, we found that when treated by the shockwave, hMSCs had decreased the chondrogenic differentiation in 3D conditions, and the mechanism was preliminarily investigated.

Shockwave can increase the concentration of extracellular adenosine. There are two main sources of extracellular adenosine. First, ATP can be released from living cells. Extracellular ATP under the action of CD73 and CD39 is gradually decomposed into ADP, AMP, adenosine^14,21^. Second, adenosine and cAMP released from the necrotic cells^22^. CD73 and prostatic acid phosphatase have been reported to convert AMP into extracellular adenosine^23,24^. Our previous study suggested that for the treatment of less than 150 impulses, there was no significant difference in cell death rate (P = 0.236); while the shockwave can promote the release of ATP in the dose-dependence manner, and the results of this study are consistent with previous findings^13^. The change trend of ADP, AMP and Adenosine was very similar to that of ATP, which indicated that the extracellular ADP, AMP and adenosine were mainly derived from the phosphohydrolysis of extracellular ATP for less than 150 impulses.

For the treatment of more than 200 impulses, the release of ATP was significantly higher compared to the control group. However, compared to the 200 group, there was no significant difference though adenosine has been shown to be on the trend of increasing. This may be explained by the treatments of increased impulse resulted the dramatic decrease of the viability of cells (p = 0.0023) followed by the decrease of the ATP synthesized by the viable cells. While the levels of cAMP and intra-cellular adenosine decreased, another source of extra cellular adenosine from nonviable cells increased.

Shockwave promotes the expression of adenosine receptor. The A3 expression in MSCs was very low, which is consistent with the literature^25^. There was no significant difference in the expressions of A2A and A3 under the treatments of shockwave. Shockwave promoted the expression of adenosine receptor of A2B. The potential mechanism may be as follows. First, the increase in the extracellular concentration of adenosine resulted in changes in the expression of adenosine receptors through a positive feedback effect. Second, shockwave treatment increased the expression of hypoxia-inducible factor-1α (HIF-1α). Analysis of the cloned human A2BR promoter identified a functional hypoxia-responsive region, including a functional binding site for HIF within the A2BR promoter. Further studies examining HIF-1α DNA binding using HIF-1α gain and loss of function experiment confirmed strong dependence of A2BR induction on HIF-1α *in vitro* and *in vivo* mouse models^26^. The increase of HIF-1α may be related with the increase in the expression of the receptor A2BR.

Shockwave inhibited the chondrogenic differentiation of hMSCs through or partially through the A2BR signaling pathway. Shockwave and adenosine inhibited transcription of SOX6 and SOX9 in the hMSCs after first two weeks of chondrogenic medium induction, further inhibiting the synthesis of COL1A2, COL2A1 and ACAN. Adenosine and specific agonist of A2B can mimic this inhibitory effect, whiles selective antagonists could reverse effects. All of the above suggested that A2BR signaling pathway mediates the inhibitory effect on the chondrogenic differentiation of hMSCs from the shockwave.

Our results also found that this may be related to IL-6. IL-6, as an inflammatory factor, might be a product of the A2B signaling pathway. Rees *et al*. reported that adenosine induced IL-6 expression in pituitary folliculostellate cells is mediated via A2BR coupled to PKC and p38 MAPK^27^. And Nakajima *et al*. showed that IL-6 can inhibit the differentiation of ATDC5 into cartilage precursor cells^28^, while it also inhibited the differentiation of hMSCs into chondrocytes in the dose-dependence manner^29^. Further evidence is needed to prove the specific mechanism of IL-6, and whether there are other factors related to it.

In this study, the chondrogenic differentiation of MSCs was inhibited by shockwave under 3D conditions. This result was consisted with Wagner *et al*. who found that High-energy ESWT might cause degenerative changes in hyaline cartilage as they were found in initial OA^30^. However, this result was different from Wang *et al*. who observed the effects of ESWT in osteoarthritis of the knee in rats^31^. Three possible reasons were as follows. First, in the current study, the negative effects of low-magnitude high-frequency vibration was testified in 3D culture *in vitro*; Second, the characteristics of the shockwave were the power and the different number of impulses; Third, all the literature have studied the shockwave effect under the condition of inflammation (such as osteoarthritis, etc.), and it is concluded that the shockwave can be beneficial to the regeneration of cartilage. But current study was based on under normal circumstances. It also suggested that there may be different effects of shockwaves in the presence of inflammatory conditions, which may be investigated in the future.

## Methods

### Ethics statement

This study was carried out in accordance with the guidelines for the care for human study adopted by the First Hospital of Jilin University, the protocol was approved by the Research Ethics Committee of the First Hospital of Jilin University (Ref. no: 2013/013), and informed consent was obtained from all participants.

### Isolation of human mesenchymal stem cells (hMSCs)

The hMSCs were isolated from human bone marrows of ten healthy voluntary donors. The marrow donors included 6 males and 4 females ranging from 21 to 49 years old. Fifteen milliliter bone marrow was aspirated from the iliac crest. Each aspirate was transferred to a 50 ml sterile tube and diluted with Dulbecco’s Modified Eagle Medium (DMEM; Hyclone) supplemented with 100 mg/mL streptomycin and 100 units/mL penicillin (1% PS; Hyclone) at ratio of 1:1. The tube was centrifuged at 600g for 10 min. The top of the fat layer was discarded, and the remaining mixture was suspended 1:1 over Ficoll (1.073 g/mL Ficoll-Plaque; GE Healthcare, Baied’Urfe, QC) and centrifuged at 900 g for 30 min. After centrifugation, the MSC enriched fraction was collected from the interface, supplemented to 40 mL with DMEM, and centrifuged at 600g for 10 min. After two washes, the cells were seeded in 75 cm^2^ tissue culture flasks and cultured in the expansion medium containing DMEM, 1% PS and 10% fetal bovine serum (FBS; Cambrex) in 5% humidified CO_2_ at 37 °C. After 48 h, the non-adherent cells were removed by medium exchange. The adhering cells were expanded in monolayer culture with medium change every 2 to 3 days until colonies reached 80% confluence. Cultures were passaged using trypsin/EDTA and reseeded at 5000 cells/cm^2^. For practical purposes, passage 2 cells in DMEM containing 20% FBS and 5% dimethyl sulfoxide were frozen in liquid nitrogen. After thawing, cells were reseeded at a density of 5000 cells/cm^2^ and passaged when 80% confluent. Passage 4 cells were used in this study.

### Shockwave Treatment

Based on our previous studies^13^, a KDE-2001 Extracorporeal Shockwave Lithotripter was used for all studies. Cells were suspended with 2ml culture medium in 15-ml sterile polystyrene tubes (Corning, USA) at a concentration of 5 × 10^5^/ml. The cells were exposed to different impulses at an energy flux density of 0.18 mJ/mm^2^. The duration of the ESWT treatment was at least 10 minutes depending on the number of impulses applied.

### Chondrogenic differentiation

For chondrogenesis in pellet cultures, isolated hMSCs (5 × 10^5^) were resuspended and spun in a 15-mL polypropylene tubes, centrifuged at 600g for 6 min and resuspended in chondrogenic medium consisting of high glucose DMEM containing 1 mM sodium pyruvate, 100 nM dexamethasone, 1% ITS + 1 (Sigma) [insulin (5 mg/mL), transferrin (5 ng/mL), and sodium selenite (5 ng/mL), 1% PS and bovine serum albumin (BSA; 1 mg/mL)], 40 μg/ml proline, and ascorbic acid (50 μg/mL) (all from Sigma-Aldrich, Poole, U.K., http://www.sigmaaldrich.com), and 10 ng/mL recombinant human transforming growth factor (TGF)-beta3 (Peprotech, Rocky Hill, NJ, USA). Cells were centrifuged at 600g for 5 min to form pellets. The pellets were cultured in a total of 1 mL chondrogenic medium per tube and incubated at 37 °C in 5% humidified CO_2_. The medium was changed every 2 to 3 days and pellets were centrifuged with every medium change.

Pellets were harvested at 7 and 21 days of culture and were washed with PBS to remove medium then transferred to RNase-free 1.5-mL micro centrifuge tubes for RNA isolation. Others were harvested at 21 days for histological and immunohistochemical analyses.

### ELISA analysis

The passage 4 cells were digested and removed to freeze storage tube at 1 ml/tube. The concentration was 5 × 10^5^/ml, and then shockwave treatment, centrifugation. Commercially available ELISA kits were used to measure supernatant concentration of ATP, AMP, ADP, adenosine IL-6 and HIF-1α(Abcam, UK). The standard procedure was followed strictly as per the kit manufacturers ELISA protocol. Instructions from the manufactures were followed.

### Histological and Immunohistochemical Analyses

Representative pellets were harvested at 21 days of culture, were rinsed with PBS, fixed in 4% paraformaldehyde, dehydrated using a graded series of ethanol washes, and embedded in paraffin. Sections 4 μm in thickness and were stained with toluidine blue.

For the immunohistochemical evaluation of cartilage-specific type II collagen, sections were incubated with primary rabbit anti-human type II collagen (1:200 dilution). The slides were treated with Multilink solution (Dako, Sydney, Australia) followed by streptavidin-conjugated peroxidase incubation. The sections were visualized with streptavidin-peroxidase Histostain SP Kit for 3, 3′-Diaminobenzidine (DAB) (Zymed Laboratories, San Francisco), and cells were counterstained with toluidine blue. Rabbit isotype IgG stained images of the Control samples were provided. Cells were examined under a Leica microscope (Leica, Heerbrugg, Switzerland, http://www.leica.com) at the indicated magnification.

### Viability assay

The cells were plated in 96-well plates at 5 × 10^3^ cells/well and allowed to grow for up to 7 days. Subsequently, the cells were incubated with 10 µl CCK-8 (Beyotime Institute of Biotechnology, Shanghai, China) at 37 °C for 1 h. The absorbance (OD) was measured in an automated microplate reader at 570 nm. Cell viability ratio was calculated with Prism5.0 software (GraphPad, La Jolla, CA/USA).

### Western blotting

Twenty-one days after chondrogenesis induction, pellets were washed in PBS, and lysed in radio-immunoprecipitation assay (RIPA) buffer with protease inhibitors (Pierce, Rockford, IL). Protein concentrations were determined using a bicinchoninic acid protein assay kit (Pierce), and an equal amount of protein from different samples was used for Western blot analysis. The samples were separated by 10% SDS–PAGE gel. These proteins were then transferred to polyvinylidene difluoride (PVDF, Millipore) membranes using a transfer unit (Bio-Rad, USA). After blocking with 5% nonfat milk, the PVDF membranes were incubated with primary antibodies in a Tris-buffered saline (TBS) buffer overnight at 4 °C. Immunoblotting was performed with antibodies against A1R, A2AR, A2BR, A3R (Cell Signaling Technology). On the following day, after washing, PVDF membranes were incubated with appropriate secondary antibodies for 2 h at room temperature. The bands were detected by ECL reagent (Thermo Fisher Scientific, Lausanne, Switzerland), the blots were visualized using X-ray film. β-Actin antibody (catalog no. A-1978; Sigma) was used as a loading control. Quantitative band-intensity analysis of Western blots was performed using CS Analyzer version 3.0 software (Atto).

### PCR

Total RNA was isolated from MSCs using TRIzol reagent (Invitrogen) and RNeasy kit (QIAGEN, Valencia, CA, http://www.qiagen.com) following manufacturers’ instructions. The purity of the RNA was assessed by measuring the A260/A280 ratio. The cDNA was prepared using reverse-transcribed with the ImProm-II reverse transcription system (Promega, Madison, WI, http://www.promega.com) from 1 mg of total RNA. The 1:40 diluted cDNA was used in 20 ml reactions for real-time PCR analysis using a Rotor-Gene RG3000 system (Corbett Life Science, Sydney, Australia). All primers were designed according to published mRNA sequences, including (1) Aggrecan F: 5′-TGA CCA CTT TAC TCT GGG TTT TCG -3′, R: 5′-ACA CGA TGC CTT TCA CCA CG-3′; (2) COL II F: 5′-CCG CGG TGA GCC ATG ATT CG-3′, R: 5′-CAG GCC CAG GAG GTC CTT TGG G-3′; (3) SOX-9 F: 5′-CACACAGCTCACTCGACCTTG-3′,R: 5′-TTCGGTTATTTTTAGGATCATCTCG-3′; (4) COL I F: 5′-GCTGGCAGCCAGTTTGAATATAAT-3′, R: 5′-CAGGCGCATGAAGGCAAGT-3′; (5) GAPDH F: 5′-ACC ACA GTC CAT GCC ATC AC-3′, R: 5′-TCC ACC ACC CTG TTG CTG TA-3. The thermal profile was as follows: 1 cycle of 95 °C for 5 min, followed by 40 amplification cycles of 95 °C for 15 s, 60 °C for 30 s, and 72 °C for 30 s. Data were analyzed by Rotor-Gene 6.0 software. Relative expression levels were calculated as a ratio to the average value of control group (MSCs during chondrogenesis which were not subjected to shockwave or indicated agents or exogenous adenosine).

### Statistical analysis

All quantitative data were expressed as mean ± standard deviation (SD) and analyzed using SPSS for Windows (SPSS version 16.0, SPSS Inc, Chicago, IL). Differences between groups were evaluated by Student’s t test as indicated. Differences were considered significant at p < 0.05.
